# Mild and Asymptomatic COVID-19 Convalescents Present Long-Term Endotype of Immunosuppression Associated With Neutrophil Subsets Possessing Regulatory Functions

**DOI:** 10.3389/fimmu.2021.748097

**Published:** 2021-09-29

**Authors:** Izabela Siemińska, Kazimierz Węglarczyk, Marcin Surmiak, Dorota Kurowska-Baran, Marek Sanak, Maciej Siedlar, Jarek Baran

**Affiliations:** ^1^ Department of Clinical Immunology, Jagiellonian University Medical College, Krakow, Poland; ^2^ Department of Internal Medicine, Jagiellonian University Medical College, Krakow, Poland; ^3^ Department of Clinical Microbiology, Laboratory of Virology and Serology, University Children’s Hospital, Krakow, Poland

**Keywords:** COVID-19 convalescents, normal density neutrophils, immunosuppression, granulocyte-macrophage colony stimulating factor (GM-CSF), low-density neutrophils (LDNs), granulocytic myeloid-derived suppressor cells (PMN-MDSCs)

## Abstract

The SARS-CoV-2 infection [coronavirus disease 2019 (COVID-19)] is associated with severe lymphopenia and impaired immune response, including expansion of myeloid cells with regulatory functions, e.g., so-called low-density neutrophils, containing granulocytic myeloid-derived suppressor cells (LDNs/PMN-MDSCs). These cells have been described in both infections and cancer and are known for their immunosuppressive activity. In the case of COVID-19, long-term complications have been frequently observed (long-COVID). In this context, we aimed to investigate the immune response of COVID-19 convalescents after a mild or asymptomatic course of disease. We enrolled 13 convalescents who underwent a mild or asymptomatic infection with SARS-CoV-2, confirmed by a positive result of the PCR test, and 13 healthy donors without SARS-CoV-2 infection in the past. Whole blood was used for T-cell subpopulation and LDNs/PMN-MDSCs analysis. LDNs/PMN-MDSCs and normal density neutrophils (NDNs) were sorted out by FACS and used for T-cell proliferation assay with autologous T cells activated with anti-CD3 mAb. Serum samples were used for the detection of anti-SARS-CoV-2 neutralizing IgG and GM-CSF concentration. Our results showed that in convalescents, even 3 months after infection, an elevated level of LDNs/PMN-MDSCs is still maintained in the blood, which correlates negatively with the level of CD8^+^ and double-negative T cells. Moreover, LDNs/PMN-MDSCs and NDNs showed a tendency for affecting the production of anti-SARS-CoV-2 S1 neutralizing antibodies. Surprisingly, our data showed that in addition to LDNs/PMN-MDSCs, NDNs from convalescents also inhibit proliferation of autologous T cells. Additionally, in the convalescent sera, we detected significantly higher concentrations of GM-CSF, indicating the role of emergency granulopoiesis. We conclude that in mild or asymptomatic COVID-19 convalescents, the neutrophil dysfunction, including propagation of PD-L1-positive LDNs/PMN-MDSCs and NDNs, is responsible for long-term endotype of immunosuppression.

## Introduction

Since the beginning of 2020, the COVID-19 pandemic has affected more than 200 million people worldwide, causing over 4.5 million deaths so far. The causative agent of COVID-19 is severe acute respiratory syndrome coronavirus 2 (SARS-CoV-2), airborne transmitted between the humans (1). Many comorbidities, such as hypertension, obesity, diabetes, and other pathologies affecting the immune system are the risk factors of the severe course of COVID-19 (2). The clinical manifestations of COVID-19 are diverse and range from asymptomatic, through mild to severe disease with lung injury and respiratory distress, often followed by multiorgan failure and death (3, 4). Blood lymphopenia is one of the hallmarks of COVID-19 and its severity correlates with worse prognosis (5, 6). However, the mechanisms underlying lymphopenia, and particularly reduction of T-cell number during COVID-19, remain unclear. The lymphocytes due to a relatively low surface expression of angiotensin converting enzyme 2 (ACE2), the entry receptor for the virus (7), seem not to be its direct target (8). Lymphopenia is not subset-specific within T cells and the numbers of both CD4^+^ and CD8^+^ T cells are rapidly reduced during the virus infection. This may be caused by the cytokine storm and rapid release of IL-6, TNF-α, and IL-1 (9), subsequent thymic involution, and/or T-cell sequestration in the specific organs due to the hyperinflammation (9, 10). However, lymphopenia has been reported concurrently with onset of the clinical symptoms (6). In this context, an alternative hypothesis claims the collapse of the host protective immunity (“immunologic collapse”), leading to failure in control of viral replication and dissemination (11–14). In this scenario, an increased production of prostaglandin D_2_ by the respiratory epithelium (15) causes inhibition of the dendritic cell response *via* DP_1_ receptor signaling and/or upregulation of myeloid cells with regulatory functions, including myeloid-derived suppressor cells (MDSCs), which may be one of the mechanisms attenuating inflammatory response (16, 17). From the other side, the early expansion of MDSCs may inhibit SARS-CoV-2 antigen-specific T-cell response and predict fatal outcome (18), suggesting these cells as important players during COVID-19.

The population of MDSCs has been defined as innate bone-marrow-derived myeloid cells suppressing effector T-cell response (19), and are considered as key cellular components connecting innate and adaptive T-cell response. They are detected mainly in cancer, where their blood level correlates with disease progression (20). Increased MDSC level was also shown in viral infections (21, 22), including COVID–19 (18, 23). By phenotype and morphology assessment, three populations of MDSCs, differing in their origin, have been distinguished so far: granulocytic (PMN–MDSCs)—Lin^–^HLA–DR^low/–^CD11b^+^CD14^–^CD15^+^, monocytic (Mo–MDSCs)—Lin^–^HLA–DR^low/–^CD11b^+^CD14^+^CD15^–^, and early stage (e–MDSCs)—Lin^–^HLA–DR^low/–^CD11b^+^CD14^–^CD15^–^, all classified as immature myeloid cells with strong immunosuppressive properties (24). The MDSC subsets differ in the mechanism of action—PMN–MDSCs are mainly responsible for reactive oxygen species (ROS) production, while Mo–MDSCs possess higher expression of inducible nitric oxide synthase (iNOS), and capacity to release large amounts of nitric oxide (NO), although some common pathways, including arginase–1 (Arg1) activity and PD–L1 expression, are also relevant (25). Their function relies mainly on suppression of T–cell response, including T–cell proliferation, IFNγ production (26), and/or induction of regulatory T cells (27). The PMN–MDSCs have lower density in contrast to normal granulocytes, which typically sediment on top of erythrocytes after density gradient centrifugation, hence, they are also called immunosuppressive low–density neutrophils (LDNs) (28) or LDNs/PMN–MDSCs, due to the lack of specific markers distinguishing neutrophil subsets within LDNs (29).

The major differences between MDSC populations and corresponding mature neutrophils and monocytes had been described (30), however, recently due to the progress in resolution techniques, including high–dimensional single–cell assays and reporter–fate mapping, concerns regarding the development and activation state of MDSCs were raised, questioning previously accepted nomenclature (31).

The role of myeloid cells with regulatory activity has been indicated in SARS–CoV–2 infection, discriminating between patients with mild and severe disease (23, 32–34). In particular, LDNs were shown to emerge in severe COVID–19 patients (32), and expansion of PMN–MDSCs with Arg1 activity was associated with an increase of the disease severity (33). The role of Mo–MDSCs, although less frequent, was also documented as related to the course of SARS–CoV–2 infection (34). All of them correlated with poor T–cell response in severe COVID–19 patients (35–37). Here, we asked whether a mild or asymptomatic course of COVID–19 may also lead to consequences in the form of systemic immunosuppression and long–COVID.

## Materials and Methods

### Study Group

The study group consisted of convalescents who recovered from a mild or asymptomatic infection with SARS–CoV–2, confirmed by the positive PCR test for SARS–CoV–2 mRNA. There were 13 individuals (7 women and 6 men) in this group aged from 29 to 58 years, with no persistent symptoms or post–COVID complications, who at the time of COVID–19 diagnosis had no symptoms (*n* = 3) or had symptoms of fever, chills, fatigue, new loss of taste or smell, cough, congestion or runny nose, headache, muscle or body aches, especially orbital region pain, and sore throat (*n* = 10), related to SARS–CoV–2 infection (convalescent characterization is presented in **Supplementary Table S1**). On the day of blood sampling, all convalescents were approximately 35 (20–60) days after the first manifestations of the disease or positive result of the RT–PCR test. The control group consisted of 13 healthy subjects without SARS–CoV–2 infection in the past, with ages from 18 to 65 years. All participants were non–vaccinated against SARS–CoV–2 before blood donation. The Bioethical Committee of the Jagiellonian University approved the study (Approval no. 1072.6120.83.2020), and all subjects gave written informed consent to participate in the study.

Peripheral blood was drawn to citrate–containing tubes (10 ml) and tubes with clot activator (3 ml). The blood count was assessed by routine procedure using a hematology analyzer (Sysmex XN–350, Sysmex, Norderstedt, Germany).

### Flow Cytometry Analysis

Whole blood (100 µl) was used for T–cell subset analysis after the staining with cocktail of the following monoclonal antibodies (mAbs): anti–CD3–FITC + anti–CD8–PE + anti–CD45–PerCP + anti–CD4–APC (BD Multitest™, BD Biosciences, Franklin Lakes, NJ, USA) and anti–PD–1–BV605 mAb (BD Biosciences). The subsets of T cells were identified after the lysis of red blood cells (RBC Lysis Buffer, eBioscience™, Invitrogen, Carlsbad, CA, USA) on FACS CantoII flow cytometer (BD Biosciences, Immunocytometry Systems, San Jose, CA, USA). Populations of CD45^+^CD3^+^CD4^+^, CD45^+^CD3^+^CD8^+^, and CD45^+^CD3^+^CD4^–^CD8^–^, corresponding to CD4^+^, CD8^+^, and double–negative T cells (DNTs), respectively, were identified using FACS Diva v. 8.0.1 software (BD Biosciences).

From the remaining blood volume, mononuclear cells (PBMC) were isolated by standard Pancoll (PAN BIOTECH, Aidenbach, Germany) density gradient centrifugation. For analysis of MDSCs, PBMCs (approximately 1 × 10^6^ cells) were stained with the following mAbs: anti–CD33–PE (clone P67.6), anti–LIN–AF700 (CD3, CD19, CD56, clones UCHT1, HIB19, and B159), anti–HLA–DR–PerCP (clone L243), anti–CD11b–BV510 (clone ICR F44), anti–CD14–FITC (clone MφP9), anti–CD15–PE–Cy7 (clone HI98), anti–PD–L1–APC (clone 10F.9G2), anti–CD64–AF700 (clone 10.1), anti–CD16–PE (clone 3G8), and anti–CD66b–FITC (clone G10F5) (all from BioLegend, San Diego, CA, USA) for 20 min at 4°C. After incubation, cells were washed twice in PBS and suspended in 0.2 ml of PBS. To determine the level of non–specific staining and cell autofluorescence, the respective isotype controls and fluorescence minus one (FMO) control samples were incubated in parallel. The samples were analyzed on FACS CantoII flow cytometer (BD Biosciences) using FACS Diva v. 8.0.1 software (BD Biosciences) and FlowJo v.10 software (BD Biosciences). The Mo–MDSCs were characterized as LIN^–^HLA–DR^low/–^CD11b^+^CD14^+^CD15^–^ cells, whereas LDNs/PMN–MDSCs were characterized as LIN^–^HLA–DR^low/-^CD11b^+^CD14^–^CD15^+^ cells. The e–MDSCs were gated as LIN^–^HLA–DR^–^CD11b^+^CD14–CD15^–^ cells. The level of MDSC subsets was presented as the percentage of nucleated cells (NC) (positive for SYTO™ 9, Invitrogen, Eugene, OR, USA). Detailed gating strategy is presented in **Supplementary Figure S1**.

### Assessment of Cell Suppressive Activity

Suppressive activity of the myeloid cell subsets was analyzed in cultures with autologous T cells activated with anti–CD3 mAb (clone HIT3, 1 μg/μl) by H^3^–thymidine incorporation assay. Briefly, the FACS–purified T cells (CD3^+^) with 10% of autologous monocytes (FACS sorted CD14^+^HLA–DR^+^) were co–cultured with LDNs/PMN–MDSCs (FACS sorted HLA–DR^low/–^CD33^+^CD66b^+^CD14^–^) or NDNs, used as control (FACS–sorted CD66b^+^ from the bottom fraction after Pancoll separation and RBC lysis) at the T cells to MDSCs/NDNs ratio 2:1 (established experimentally), and activated with *anti–CD3* mAb (clone HIT3, 1μg/μl, BD Pharmingen™, Franklin Lakes, NJ, USA). After 3 days of culture, T cells were pulsed with H^3^–thymidine (1 μCi/well, GE Healthcare, Marlborough, CT, USA) for an additional 6 h and thymidine uptake was measured as counts per minute (cpm) in a liquid scintillation counter LS1801 (Beckman Coulter, Indianapolis, IN, USA). The results are presented as mitotic index:


$$\text{mitotic index}=\frac{\text{anti}-\text{CD}3\text{ stimulated test culture }[\text{cpm}]}{\text{non}-\text{stimulated culture }[\text{cpm}]}$$

### Testing for Anti–SARS–CoV–2 S1 IgG

Anti–SARS–CoV–2 S1 IgG antibodies were quantified in fresh serum samples. The level of antibodies was determined by SARS–CoV–2 S1 IgG II Quant chemiluminescent microparticle immunoassay (Abbott Laboratories, Lake Bluff, IL, USA), using “Aliniti i” immune analyzer (Abbott), following the manufacturer’s instructions.

### Evaluation of GM–CSF Concentration

Concentration of GM–CSF was assessed in serum samples stored at −20°C in one batch measurement. All samples were thawed, and the concentration of GM–CSF was evaluated by ELISA immunoassay (R&D Systems, Minneapolis, MN, USA), according to the manufacturer’s recommendations.

### Statistical Analysis

Statistical analysis was performed using the PRISM GraphPad 8 package (GraphPad Software Inc., San Diego, CA, USA). Data were analyzed using *t*–test or one–way analysis of variance (ANOVA) with Tukey Multiple Comparison Test, as a *post–hoc* test. The magnitude of the relationship between two quantitative features was evaluated using Pearson’s correlation coefficient. All data are expressed as median with interquartile range. *p* < 0.05 was considered statistically significant.

## Results

### PBMCs From COVID–19 Convalescents Contain High Frequency of LDNs/PMN–MDSCs

**Figure 1:**
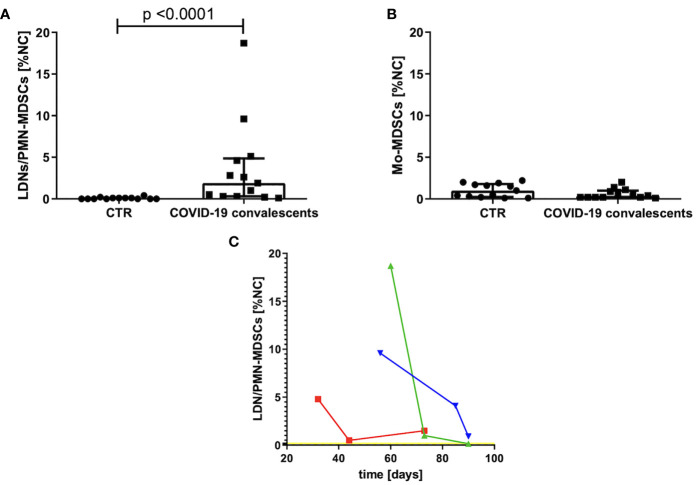
**(A)** Frequency of LDNs/PMN–MDSCs in the blood of convalescents and healthy controls (CTR). MDSCs were identified by flow cytometry after gating according to the cell surface antigens, as described in *Materials and Methods* and presented in **Supplementary Figure S1**. Cell frequency was calculated as percentage of nucleated cells (NC) from PBMCs. **(B)** Frequency of Mo–MDSCs in the blood of convalescents and healthy controls (CTR). MDSCs were identified by flow cytometry after gating according to the cell surface antigens, as described in *Materials and Methods* and presented in **Supplementary Figure S1**. Cell frequency was calculated as percentage of nucleated cells (NC) from PBMCs. **(C)** Changes in LDNs/PMN–MDSCs frequency in peripheral blood as a function of time after COVID–19 recovery. Around day 80 from COVID–19 infection, the level of LDNs/PMN–MDSCs is dropping noticeably in the blood of convalescents. LDNs/PMN–MDSCs level was analyzed in three time points by flow cytometry as LIN^–^HLA–DR^low/–^CD11b^+^CD14^–^CD15^+^ cells and calculated as percentage of nucleated cells (NC) from PBMC. Data from three patients with initial highest level of LDNs/PMN–MDSCs are presented. The average level of LDNs/PMN–MDSCs in peripheral blood of healthy donors is indicated by a yellow line.

The group of convalescents was examined, on average, after 35 days (20–60 days) from the first symptoms or, in the case of asymptomatic course, from the day of positive result of the RT–PCR test for SARS–CoV–2 mRNA. The MDSCs were identified as Lin^–^HLA–DR^low/–^CD11b^+^ cells and further divided into Mo–MDSCs, LDNs/PMN–MDSCs, and e–MDSCs, based on the expression of CD14 and CD15. Their frequency was evaluated in PBMCs of convalescents and compared with the age–matched healthy controls (CTR). Level of e–MDSCs was negligible (data not shown), whereas the frequency (percent value of NC) of Mo–MDSCs in the study group did not differ from healthy controls (**Figure 1B**). In contrast, frequency of LDNs/PMN–MDSCs was significantly higher in PBMCs of convalescents in comparison to healthy controls (*p* < 0.0001) (**Figure 1A**).

Therefore, in the follow–up, we focused on LDNs/PMN–MDSCs only. An elevated level of these cells had reduced individually with various rates, in three cases with the highest frequency, it reached the level of healthy donors after ca. 3 months from infection (**Figure 1C**).

### Composition of the Main Subsets of T Cells Is Altered in Peripheral Blood of COVID–19 Convalescents and Includes High Level of CD8^+^ T Cells With Phenotype of Exhaustion

**Figure 2:**
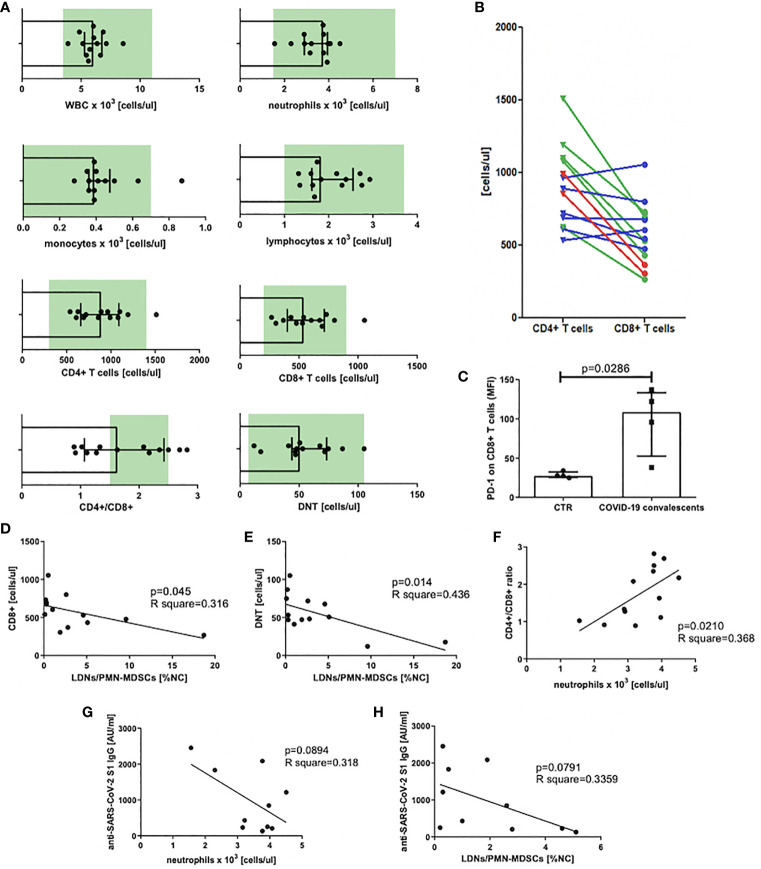
**(A)** Blood WBC, leukocyte populations, and T–cell subset count in peripheral blood of COVID–19 convalescents with normal range marked in green. Normal range for DNTs was calculated from T cells [1%–5% of T cells, (38)] (*n* = 13). **(B)** CD4^+^ and CD8^+^ T–cell distribution in individual patients. Patients with CD4^+^/CD8^+^ ratio above the normal range are marked in red, within the norm in green, and below the norm in blue (*n* = 13). **(C)** PD–1 expression on CD8^+^ T cells of COVID–19 convalescents. Expression of PD–1 on CD8^+^ T cells of COVID–19 convalescent and healthy controls was evaluated by flow cytometry and presented as mean fluorescent intensity (MFI). Data from four subjects in each group are presented (*n* = 4). **(D)** Correlation of the level of LDNs/PMN–MDSCs and the count of CD8^+^ in blood of COVID–19 convalescents. CD8^+^ T cells were identified by flow cytometry as CD45^+^CD3^+^CD8^+^ cells and their concentrations were calculated from the total lymphocyte counts (*n* = 13). **(E)** Correlation of the level of LDNs/PMN–MDSCs and the count of double–negative T cells (DNT) in blood of COVID–19 convalescents. Double–negative T cells (DNTs) were identified by flow cytometry as CD45^+^CD3^+^CD4^–^CD8^–^ and their concentrations were calculated from the total lymphocyte counts (*n* = 13). **(F)** Correlation of CD4^+^/CD8^+^ T cell ratio with neutrophil count in blood of COVID–19 convalescents (*n* = 13). **(G)** Correlation of anti SARS– CoV–2 S1 IgG antibody concentration with the number of neutrophils in COVID–19 convalescents (*n* = 10). **(H)** Correlation of anti–SARS CoV–2 S1 IgG antibody concentration with the frequency of LDNs/PMN–MDSCs in COVID–19 convalescents (*n* = 10).

At first, we analyzed the count of white blood cells (WBCs) and specific leukocyte populations, including neutrophils, monocytes, lymphocytes, T cells, and their main subsets, namely, CD8^+^, CD4^+^, and DNTs in peripheral blood of COVID–19 convalescents. This analysis revealed that WBC counts already normalized in all individuals at the time of the study. Similarly, blood counts of neutrophils, monocytes (except one case), lymphocytes, T cells, and their subsets, except one case, were in normal ranges. In some individuals the mean value of the CD4^+^/CD8^+^ ratio significantly differed from the reference interval after recovery from COVID–19 (**Figure 2A**). In the case of six convalescents, this parameter was still less than 1.5, in two cases, it was even less than 1.0, and in two other cases, it was higher than 2.5 (**Figure 2A**). These data indicate that in majority of the convalescents, CD8^+^ T cells were induced by SARS–CoV–2 (**Figure 2B**). However, these CD8^+^ T cells when further analyzed, showed to be positive for PD–1 expression (**Figure 2C**), suggesting their exhaustion due to stimulation by viral antigens. In parallel, we correlated the absolute numbers of circulating CD8^+^, CD4^+^, and DNTs with the level of LDNs/PMN–MDSCs. This analysis showed that the numbers of CD8^+^ T cells and DNTs negatively correlated with the frequency of PMN–MDSCs (**Figures 2D, E**). In respect to CD4^+^ T cells, such an association was not observed (data not shown). Moreover, we have noticed a positive correlation between the CD4^+^/CD8^+^ ratio and the number of circulating neutrophils (**Figure 2F**).

### LDNs/PMN–MDSCs and NDNs May Affect Anti–SARS–CoV–2 Antibody Production

In the next step, we asked if LDNs/PMN–MDSCs may impact on anti–SARS–CoV–2 antibody production. To address this question, we evaluated the serum level of anti–SARS–CoV–2 S1 IgG antibodies and correlated with the frequency of LDNs/PMN–MDSCs in blood of COVID–19 convalescents. The obtained results showed a clear tendency for negative correlation between these two parameters. Additionally, similar dependency was observed in relation to neutrophils. Although these data did not reach a statistical significance (**Figures 2G, H**), the tendency suggests that LDNs/PMN–MDSCs and NDNs interfere with anti–SARS–CoV–2 antibody production in COVID–19 convalescents.

### Both LDNs/PMN–MDSCs and NDNs From COVID–19 Convalescents Possess Immunosuppressive Activity

**Figure 3:**
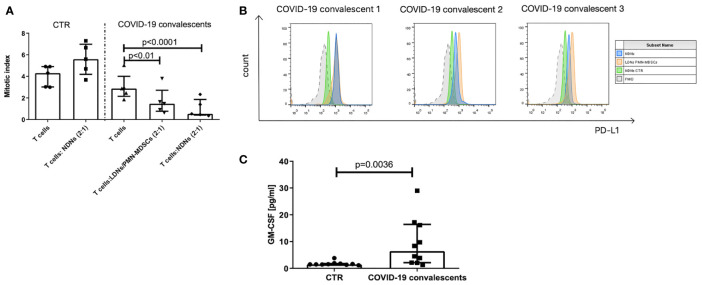
**(A)** LDNs/PMN–MDSCs and NDNs from COVID–19 convalescents inhibit proliferation of autologous T cells. T cells were stimulated with anti–CD3 mAb for 3 days in the presence of LDNs/PMN–MDSCs or NDNs. LDNs/PMN–MDSCs were isolated by FACS as HLA–DR^low/–^CD33^+^CD66b^+^CD14^–^ cells from PBMCs, while NDNs were sorted out as CD66b^+^ cells from the pellet obtained by density gradient centrifugation and RBC lysis. LDNs/PMN–MDSCs and NDNs were added to the culture of FACS purified autologous CD3^+^ T cells with 10% of autologous monocytes (sorted CD14^+^ HLA–DR^+^ cells). After 3 days of co–culture, T cells were pulsed with H^3^–thymidine for an additional 6 h and β^–^ radiation was measured as cpm in a liquid scintillation counter. The index of proliferation was calculated as a ratio of anti–CD3 stimulated test culture [cpm] and non–stimulated culture [cpm] (*n* = 5). **(B)** PD–L1 expression on LDNs/PMN–MDSCs (orange) and NDNs (blue) of COVID–19 convalescent and healthy control (green). LDNs/PMN–MDSCs were gated as presented in **Supplementary Figure S1**. Individual data from three convalescents are shown. **(C)** Serum concentration of GM–CSF in COVID–19 convalescents and healthy controls (*n* = 10).

Identification of myeloid cells with regulatory activity requires, in addition to their immunophenotype characterization, also a confirmation of their suppressive nature (30). Following this requirement, the FACS–sorted LDNs/PMN–MDSCs from the blood of COVID–19 convalescents were added to the cultures of autologous anti–CD3–stimulated T cells. Simultaneously, as controls, anti–CD3–stimulated T cells from COVID–19 convalescents and healthy donors were cultured alone or in the presence of autologous NDNs, added at the same ratio. The obtained results showed that T cells from COVID–19 convalescents already have a tendency (n.s.) for diminished proliferation ability in response to stimulation (**Figure 3A**). These data, although not statistically significant, collaborate the results by others (39). Moreover, LDNs/PMN–MDSCs from the COVID–19 convalescents effectively suppressed anti–CD3–induced proliferation of autologous T cells (*p* < 0.01). Surprisingly, NDNs from the convalescents also showed suppressive activity, and this was even more pronounced compared to LDNs/PMN–MDSCs (*p* < 0.0001). On the contrary, NDNs from healthy donors’ blood did not have such activity, instead, they slightly enhanced proliferation of autologous T cells stimulated with anti–CD3 mAb (**Figure 3A**).

Searching for the reason of suppression induced by neutrophil subsets from COVID–19 convalescents, we looked at the PD–L1 expression as a key marker related with such an activity and found that both populations of LDNs/PMN–MDSCs and NDNs showed high expression of immunosuppressive PD–L1 (**Figure 3B**). Further, we took an advantage from the study by Khanna et al., who described a similar phenomenon operating in mesothelioma patients, and pointed on GM–CSF as a factor promoting emergency myelopoiesis and granulocyte–related immunosuppression (40). With this in mind, we analyzed the GM–CSF level in the convalescent’s sera. Results from the ELISA measurements showed that sera from COVID–19 convalescents contain significantly higher concentration of GM–CSF than sera from healthy donors (**Figure 3C**).

## Discussion

SARS–CoV–2 infection is associated with lymphopenia and profound alterations of the myeloid compartment (32, 39). Here we showed dysfunctions of myeloid cells also in convalescents from mild/asymptomatic COVID–19. Specifically, our data suggest that propagation of LDNs/PMN–MDSCs and presence of NDNs with regulatory functions are responsible for long–term endotype of immunosuppression in this group. Recently, in the elegant study, Schulte–Schrepping et al. using scRNA–seq analysis showed in–depth COVID–19–associated alterations in monocyte and neutrophil components, documenting occurrence of immature and dysfunctional neutrophils and HLA–DR^low^ monocytes during the severe course of disease (35). Accumulation of HLA–DR^low^ monocytes, suggesting an impairment of antigen presentation to naive T cells in severe form of infection, has also been detected by others (3, 41–44). HLA–DR downregulation, typical for MDSCs, was also shown to immediately precede progression to severe respiratory failure (45). In opposite to severe COVID–19, in patients with mild course of infection, the HLA–DR^high^/CD11c^high^ inflammatory monocytes with an interferon–stimulated gene signature were detected (35). In our study, 1 month after infection, we observed no difference in HLA–DR expression level on monocytes, comparing convalescents to healthy donors (**Supplementary Figure S2A**), suggesting that such cells might be only temporarily present in peripheral blood. Also, Mo–MDSCs were found to expand in blood of COVID–19 patients and associate with disease severity (36), however, in the case of convalescents, we did not detect changes in the frequency of this cell subset in PBMCs, when comparing to healthy controls (**Figure 1**).

In respect to neutrophils, they comprise a heterogenous cell population, differing both in their functions and density. In pathological conditions, including infections, the presence of LDNs within the fraction of mononuclear cells in the interphase after density gradient centrifugation has been reported (28, 46), with a substantial composition of immunosuppressive PMN–MDSCs (47). Neutrophils upon activation and degranulation secrete arginase–1 and produce ROS to mediate cell suppression, indicating functional and phenotype overlap with PMN–MDSCs (48–50). In line with this, many studies have described LDNs as being composed of immature neutrophils (51), heterogeneous populations consisting of both immature and mature “neutrophil–like” populations (52, 53), and “activated/degranulated” mature neutrophils (49, 54, 55).

Several markers, including maturation, e.g., CD10, CD11b, and CD16, or activation ones, e.g., CD66b, CD64, PD–L1, CD11b, and CD16, have been proposed to differentiate neutrophil subsets in LDNs, depending on the study design (56–58). However, recent sc–RNAseq analysis in severe and mild COVID–19 patients revealed the presence of seven phenotypically distinct neutrophil clusters within LDNs (35). With this in mind, to indicate the complexity of LDNs, in the current paper, we have used the previously proposed acronym LDNs/PMN–MDSCs (28).

In the case of SARS–CoV–2 infection, myeloid cells with regulatory functions, including those of neutrophil origin, have been studied so far, mainly in terms of their effect on the duration and course of disease (23, 36, 59, 60). In this context, it has been shown that early expansion of these cells may predict a fatal outcome of COVID–19 (18) and their level is higher in patients with severe course of infection (61). In line with this, MDSCs have been postulated as a potential biomarker and therapeutic target in COVID–19 (23). Here, we have shown that the frequency of LDNs/PMN–MDSCs is increased in the convalescents’ blood even 2–3 months from mild or asymptomatic infection (e–MDSCs were not detectable at this time). A higher level of PMN–MDSCs, compared to healthy donors, was already observed in patients with mild course of disease (61), while delayed and transient expansion of this cell subset in the cohort of severely ill Japanese patients was correlated with inhibition of the harmful immune response (34). These authors even proposed the level of PMN–MDSCs as a prognostic factor for severe COVID–19 patients. At the same time, they did not notice any increase in this cell subset level in cases of mild course of COVID–19. This discrepancy may result from the use of frozen PBMCs in the abovementioned study, not recommended for the detection of this type of cells (62). In the case of our study group, decrease in the frequency of LDNs/PMN–MDSCs in PBMCs after infection was slow and was close to the level of healthy donors after ca. 3 months of infection. To the best of our knowledge, this is the first study documenting such an observation in convalescents after mild or asymptomatic infection. In the case of convalescents, we did not observe, typical for acute COVID–19 lymphopenia (63, 64), which is associated with severe disease (6, 65) and usually is reversed when patients recover (6, 66). In some patients, lymphopenia has been reported to affect CD4^+^ and CD8^+^ T cells, and other lymphocytes (12, 45, 66), whereas many data suggest that SARS–CoV–2 infection has a preferential impact on CD8^+^ T cells (63, 67). In this context, CD8^+^ T cells from our convalescents, although in a normal range for their absolute number, were positive for PD–1, known as a T–cell exhaustion marker (68). Functional exhaustion of T cells during COVID–19 has been already documented by many groups (12, 14, 37, 69).

Zheng et al. reported that elevated exhaustion levels and reduced functional diversity of T cells in peripheral blood may predict the disease progression (13). However, expression of the exhaustion markers could also reflect recent activation, and it is not clear whether T cells in patients with COVID–19 are exhausted or just highly activated (69). In keeping, some reports question the exhaustion of PD–1^+^ cells in COVID–19, suggesting that PD–1^+^ T cells are fully functional in these patients (70, 71). In our experimental settings, we did not assess function of CD8^+^ T cells, however, analysis of their concentration in peripheral blood of convalescents clearly showed a negative correlation between the two. This indirectly supports hypothesis on the regulatory effect of LDNs/PMN–MDSCs on CD8^+^ effector T cells in COVID–19 convalescents. Importantly, a drop in CD8^+^ level was associated with the severe course of disease, and posttreatment decrease in CD8^+^ T cells and increase in CD4^+^/CD8^+^ ratio were indicated as independent predictors of poor effectiveness of therapy (71, 72).

We have noticed the imbalance in the CD4^+^/CD8^+^ T cell ratio, which normally oscillates between 1.5 and 2.5 (72). In 46% of convalescents, this ratio was below 1.5. Such disturbances were already observed in COVID–19 patients (64). Interestingly, our research showed a strong association between CD4^+^/CD8^+^ ratio and neutrophil count, suggesting that cells of granulocyte origin may have an impact on this parameter, most likely affecting frequency of CD8^+^ T cells. In the work by Li et al., the neutrophil count and CD4^+^/CD8^+^ ratio were among the top five variables contributing in mild COVID–19 cases, selected using a machine learning approach (69).

Suppression of CD8^+^ T cells by MDSCs is well documented and one of the mechanisms involved is the production of immunosuppressive cytokines, mainly TGF–β and IL–10 (73). In the case of SARS–CoV–2 infection, the decrease of blood level of DNTs was also negatively correlated with IL–10 (74). DNTs contribute to inflammation and were found to act as regulatory and/or cytotoxic T cells (75). Given that the level of DNTs decreases in the initial stage of infection and it correlates with fever in COVID–19 (76), these cells can be assigned a cytotoxic role in SARS–CoV–2 infection. Additionally, MDSCs constitute a source of IL–10 (73) and the level of IL–10 is elevated in COVID–19 patients (12). In a current study, we have shown a negative correlation between DNTs count and frequency of LDNs/PMN–MDSCs. This observation indirectly supports previous data on the role of IL–10 in reducing the number of DNTs (74) and suggests such a mechanism operating in COVID–19.

Although humoral immune response may also be hampered by MDSCs (77), there is no direct evidence on the diminished antibody production by B cells during COVID–19 (37). In our study, we have shown a tendency for negative correlation between LDNs/PMN–MDSC level and the concentration of anti–SARS–CoV–2 neutralizing IgG antibody in the convalescents after 20–60 days from infection. In line with this, it was documented that the level of the spike protein–specific memory B cells increases around 30–60 days after infection (78). Although we have no formal proof, it is tempting to speculate that this might be accompanied by normalization of the LDNs/PMN–MDSCs level observed in our study. Tentatively, the phenomenon could be explained by antibody–dependent enhancement if complexes of neutralizing antibodies and viral antigens were bound to FcγRII. This aspect, however, needs to be further investigated.

While the suppressive nature of LDNs/PMN–MDSCs is not surprising, detection of the similar activity of NDNs was unexpected. In our experimental settings, NDNs from COVID–19 convalescents exhibited robust suppressive activity on proliferation of autologous T cells, and this was even stronger than mediated by LDNs/PMN–MDSCs. Such NDNs have been already described in cancer patients (40, 79) and patients with severe COVID–19, where the presence of dysfunctional neutrophils, including LDNs, was linked to emergency myelopoiesis (35). Several studies have identified emergency myelopoiesis as a hallmark of fatal COVID–19 (42, 80) and particularly neutrophil counts were found to be significantly elevated in patients with COVID–19 and correlated with disease severity (81, 82). In this context, it is worth mentioning that in differential analysis, both LDNs/MN–MDSCs and NDNs are counted as peripheral blood neutrophils, affecting the neutrophil–to–lymphocyte ratio in COVID–19 patients. The use of HLA–DR, CD16, CD64, and CD66b markers was able only to indicate the presence of activated neutrophils within the LDNs/PMN–MDSCs (slight increase in HLA–DR and CD66b expression, and presence of CD64^high^ cells) and more mature CD16^+^ subset within NDNs but did not allow one to precisely distinguish the composition of the two. In respect to HLA–DR, its expression on LDNs/PMN–MDSCs (characteristic feature of these cells) of convalescents seems to further decrease over time (**Supplementary Figure S2**).

Interestingly, neutrophils from patients with severe SARS–CoV–2 infection featured expression of genes related to suppressive functionality, including *ARG1* and *CD274* (PD–L1) (83), while culture supernatants from neutrophils isolated from COVID–19 patients inhibited T–cell proliferation (84). It was also postulated that activated neutrophils may exert myeloid–derived suppressor cell activity (48). In our study, both LDNs/PMN–MDSCs and NDNs were positive for the surface expression of PD–L1, indicating its role in direct cell–to–cell mediated immunosuppression of T–cell response. This, however, does not exclude the involvement of other suppressive–like molecules released by these cell subsets and operating in COVID–19 patients, e.g., arginase–1 or ROS (22, 33, 85). This mechanism could explain a more potent suppressive nature of NDNs despite their generally lower expression of PD–L1, compared to LDNs/PMN–MDSCs. Altogether, our data suggest that in case of COVID–19 convalescents, NDNs and LDNs/PMN–MDSCs may differ in activation/maturation status and mechanism of suppressive activity, with a common pathway involving PD–L1 expression. However, whether and to what degree NDNs display properties like those described for LDNs is still unclear (29).

The mechanism responsible for neutrophil dysfunction further leading to T–cell suppression may be related to GM–CSF activity (40). GM–CSF is an emergency myelopoiesis cytokine and may induce neutrophil (hyper)–activation and degranulation (86) through STAT3 phosphorylation (87). In addition, both GM–CSF and STAT3 are associated with the induction of neutrophils with regulatory functions (88). In this context, an increased GM–CSF concentration in convalescents observed in our study, also shown by others in COVID–19 patients (89), may be responsible for neutrophil (hyper)–activation and induction of their suppressive activity. In this context, we cannot exclude the role of other cytokines, e.g., IL–6, TNF–α, and IL–1, responsible for “cytokine storm” associated with several detrimental clinical features of COVID–19 in patients with severe course of disease. In the case of convalescents from mild/asymptomatic COVID–19, these cytokines (in significantly lower concentrations) could be involved in secondary activation of “immature” myeloid cells, further developing their regulatory functions (90). Recent data by Chu et al. showing no difference in serum cytokine concentrations between the mildly and more severely affected COVID–19 patients 6 weeks after infection seem to support this scenario (91), however, the levels of respective cytokines were not compared to healthy subjects.

In conclusion, although our group of subjects was small, we were able to show that in convalescents from COVID–19 after 2–3 months from infection, both LDNs/PMN–MDSCs and NDNs possess immunosuppressive properties against T cells. Transient expansion of LDNs/PMN–MDSCs and dysfunction of NDNs after asymptomatic and mild course of SARS–CoV–2 infection may be caused by GM–CSF production and upregulation of PD–L1 expression, leading to prolonged immunosuppression in COVID–19 convalescents. However, in the light of the current controversy in definition of myeloid regulatory cell populations (31), the precise origin of immunosuppressive LDNs, altered granulopoiesis, and/or regulatory properties acquired by NDNs in response to SARS–CoV–2 infection and long–COVID remains to be determined.

## Data Availability Statement

Upon reasonable request, the raw data supporting the results of this article will be made available by the authors without undue reservation.

## Ethics Statement

The studies involving human participants were reviewed and approved by Bioethical Committee of the Jagiellonian University. The patients/participants provided their written informed consent to participate in this study.

## Author Contributions

IS and JB conceived the study. IS performed most of the experiments, analyzed data, and wrote the draft version of the manuscript. KW performed FACS sorting. MSu performed GM–CSF analysis. DK–B performed anti–SARS–CoV–2 S1 IgG testing. MSa and JB contributed to funding acquisition. MSa and MSi revised the manuscript. JB and IS wrote the final version of the manuscript. All authors contributed to the article and approved the submitted version.

## Funding

The authors declare and acknowledge financial support from project grant 19/WFSN/2020 and 35/WFSN/2020 from the Ministry of Science and Higher Education in Poland and from the EU H2020–MSCA–RISE–2016 program – grant “CHARMED” (GA 734684).

## Conflict of Interest

The authors declare that the research was conducted in the absence of any commercial or financial relationships that could be construed as a potential conflict of interest.

## Publisher’s Note

All claims expressed in this article are solely those of the authors and do not necessarily represent those of their affiliated organizations, or those of the publisher, the editors and the reviewers. Any product that may be evaluated in this article, or claim that may be made by its manufacturer, is not guaranteed or endorsed by the publisher.
