# Supplementary material for: Mild and Asymptomatic COVID-19 Convalescents Present Long-Term Endotype of Immunosuppression Associated With Neutrophil Subsets Possessing Regulatory Functions
Source: Front Immunol. 2021 Sep 29;12:748097. doi: 10.3389/fimmu.2021.748097 (PMC8511487; doi:10.3389/fimmu.2021.748097)
Supplement: Supplementary file 1 [file DataSheet_1.docx]

Supplementary Material

**Table.1**

Clinical characterization of the COVID-19 convalescents.

| Lp. | Age | Sex | BMI | WHO COVID-19 classification | comorbidities | medication | smoking history |
| --- | --- | --- | --- | --- | --- | --- | --- |
| 1 | 37 | W | 18,96 | 1 | - | glucocorticosteroids | Never |
| 2 | 46 | W | 20,83 | 1 | - | - | Never |
| 3 | 29 | W | 21.26 | 1 | - | paracetamol | Never |
| 4 | 55 | M | 25.56 | 1 | - | paracetamol | Never |
| 5 | 56 | M | 25.83 | 1 | hypertension | glucocorticosteroids, ceftriaxone | ex-smoker |
| 6 | 35 | M | 23.72 | 1 | - |  | Never |
| 7 | 29 | M | 25.06 | 1 | - | - | current |
| 8 | 29 | W | 18.56 | 1 | - | paracetamol | Never |
| 9 | 42 | M | 21.97 | 0 | - | - | Never |
| 10 | 43 | W | 18.42 | 0 | - | - | Never |
| 11 | 58 | M | 25.04 | 1 | hypertension | paracetamol | Never |
| 12 | 34 | W | 19.92 | 0 | - | - | Never |
| 13 | 47 | W | 20.09 | 1 | - | - | never |

Grey area marks patients for which longitudinal samples (n=3) were collected and analyzed.


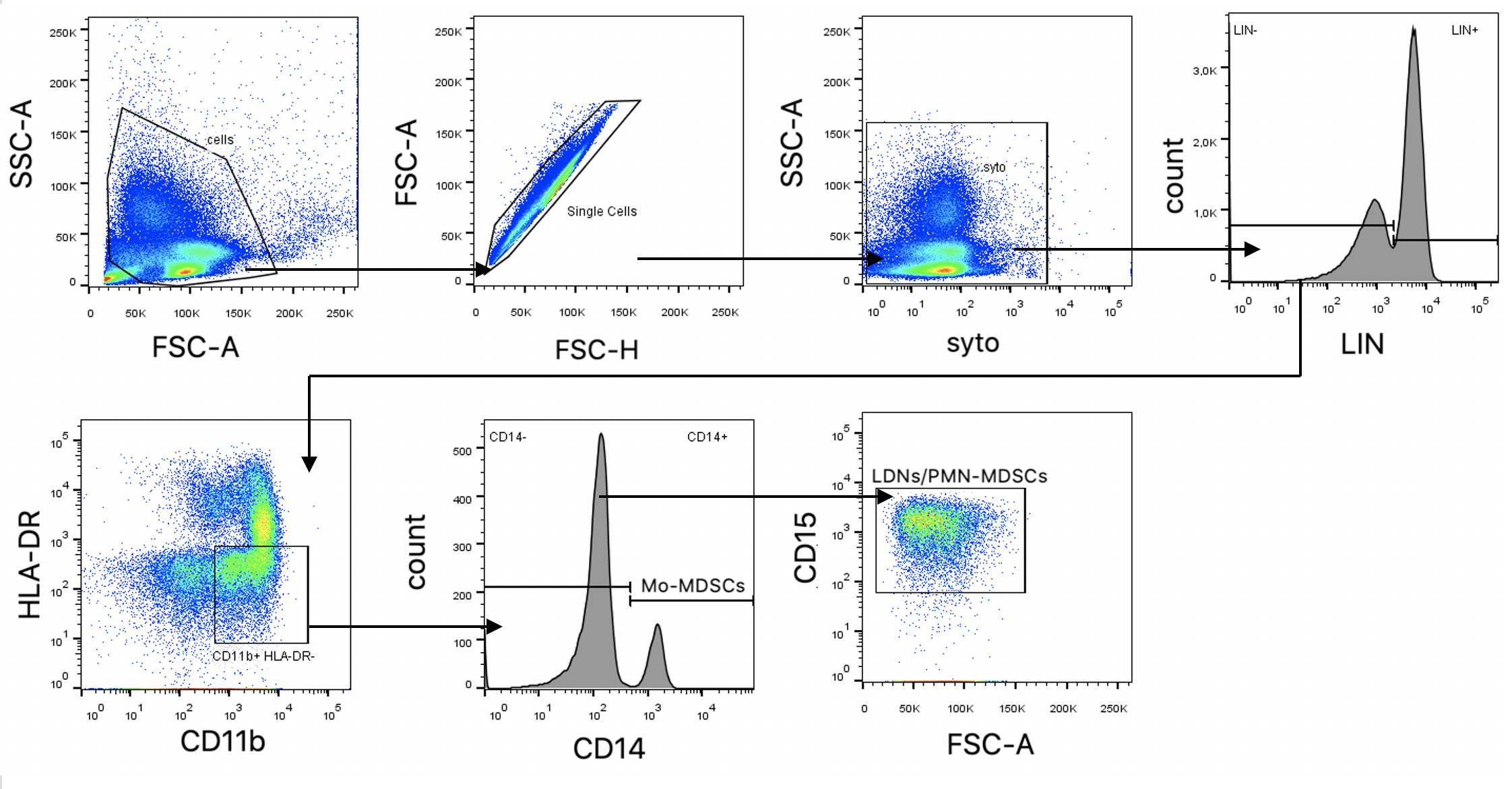


**Supplementary Figure 1.**

Gating strategy. The LDNs/PMN-MDSCs were gated as LIN^-^ from singlet nucleated cells (SYTO 9-positive), then CD11b^+^HLA- DR^low/-^ cells were selected and Mo-MDSCs were identifies as CD14^+^, LDNs/PMN-MDSCs were identified as CD14^-^CD15^+^ cells.


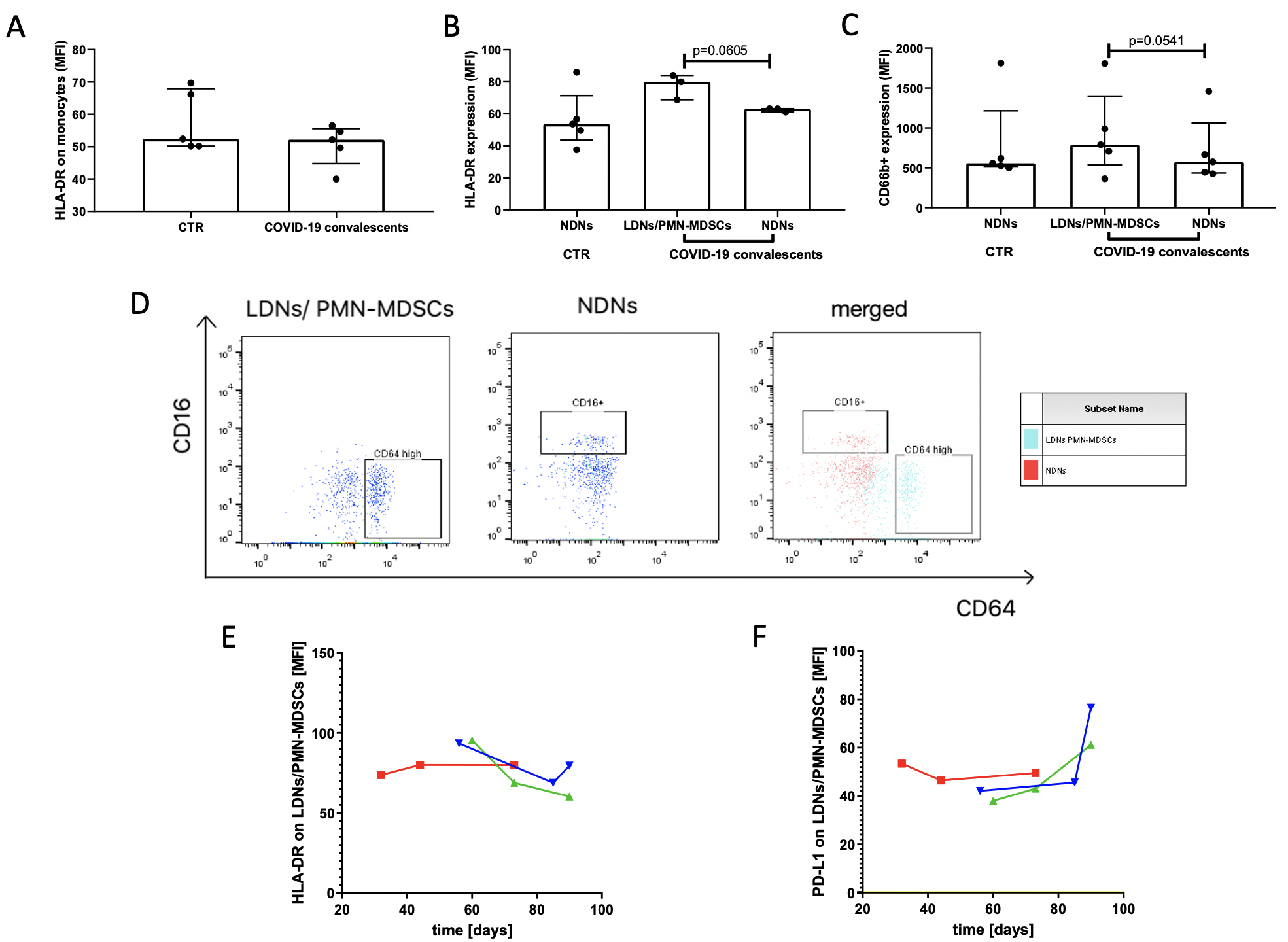


**Supplementary Figure 2.**

1. HLA-DR expression on monocytes in COVID-19 convalescents and healthy controls. Expression of HLA-DR on monocytes (gated as Lin^-^CD11b^+^CD14^+^) was evaluated by flow cytometry and presented as MFI. Data from five subjects in each group are presented (n=5).
2. HLA-DR expression on LDNs/PMN-MDSCs and NDNs in COVID-19 convalescents and healthy controls. Expression of HLA-DR on LDNs/PMN-MDSCs (gated as described in Supplementary Figure 1) and NDNs was evaluated by flow cytometry and presented as MFI. Data from five HD (n=5) and three convalescents are shown (n=3).
3. CD66b^+^ expression on LDNs/PMN-MDSCs and NDNs in COVID-19 convalescents. Expression of CD66b+ on LDNs/PMN-MDSCs (CD33^+^HLA-DR^low/-^CD14^-^CD66b^+^ cells in PBMC) and NDNs (CD66b^+^ from the bottom fraction after Pancoll density gradient separation and RBC lysis) was evaluated by flow cytometry and presented as MFI. Data from five subjects (n=5) are presented.
4. Comparison of CD16 and CD64 expression on LDNs/PMN-MDSCs and NDNs. Data from one representative flow cytometry analysis are shown.
5. Changes in HLA-DR expression on LDNs/PMN-MDSCs in COVID-19 convalescents. Expression of HLA-DR (MFI) on LDNs/PMN-MDSCs from convalescents (n=3) corresponding to Figure 1C is presented.
6. Changes in PD-L1 expression on LDNs/PMN-MDSCs in COVID-19 convalescents. Expression of PD-L1 on LDNs/PMN-MDSCs (gated as presented in Figure 1) was evaluated by flow cytometry and presented as MFI. Data from three subjects (n=3) corresponding to Figure 1C is presented.
